# Cavitation and Solid-State Post-Condensation of Polyethylene Terephthalate: Literature Review

**DOI:** 10.3390/ma17225637

**Published:** 2024-11-18

**Authors:** Paweł Wawrzyniak, Waldemar Karaszewski, Artur Różański

**Affiliations:** 1 Faculty of Automotive and Construction Machinery Engineering, Warsaw University of Technology, 84 Ludwika Narbutta Street, 02-524 Warsaw, Poland; 2 Faculty of Mechanical Engineering and Ship Technology, Gdańsk University of Technology, 11/12 Gabriela Narutowicza Street, 80-233 Gdańsk, Poland; waldemar.karaszewski@pg.edu.pl; 3 Centre of Molecular and Macromolecular Studies, Polish Academy of Sciences, 112 Sienkiewicza Street, 90-363 Łódz, Poland; artur.rozanski@cbmm.lodz.pl

**Keywords:** PET, cavitation, solid-state post-condensation, positron annihilation lifetime spectroscopy (PALS)

## Abstract

Polyethylene terephthalate (PET) is widely used in bottle production by stretch blow molding processes (SBM processes) due to its cost-effectiveness and low environmental impact. The presented literature review focuses on microcavitation and solid-state post-condensation effects that occur during the deformation of PET in the SBM process. The literature review describes cavitation and microcavitation effects in PET material and solid-state post-condensation of PET on the basis of a three-phase model of the PET microstructure. A three-phase model of PET microstructure (representing the amorphous phase in two ways, depending on the ratio of the trans-to-gauche conformation of the PET macromolecule and the amount of free volume) with a nucleation process, a crystallization process, and the use of positron annihilation lifetime spectroscopy (PALS) to analyze PET microstructure are discussed in detail. The conceptual model developed based on the literature combines solid-state post-condensation with microcavitation via the diffusion of the post-condensation product. This review identifies the shortcomings of the developed conceptual model and presents them with five hypotheses, which will be the basis for further research.

## 1. Introduction

The presented literature review focuses on microcavitation and solid-state post-condensation effects that occur during the deformation of PET in the stretch blow molding (SBM) process. Other articles comprehensively review the SBM process using cold and hot molds [1,2,3,4,5,6]. The microcavitation process occurring in the SBM process was first observed by the author of this review in 2020 [5,6]. However, the solid-state post-condensation effect occurring in the SBM process was first observed by the author of this review in 2024 [7,8].

In one study [7], an investigation of the effects of recycled PET (rPET) content and preform heating and cooling conditions in SBM processes on the microscopic properties of bottles (including crystallinity, density, viscosity, relaxation degree of the amorphous phase, and microcavitation) was described in detail, and this literature review is the basis for the statistical interpretation of the research results presented in work [8]. Drawing conclusions from statistical analysis requires holistic knowledge about the microstructure of PET, and that is why the literature review of the microstructure of PET, the measurements of this microstructure—which are also based on positron annihilation lifetime spectroscopy (PALS), cavitation and microcavitaion, and the solid-state post-condensation (SSPC) of PET—was prepared.

In the literature review, first, the concept of a three-phase PET model, the crystal nucleation process based on the three-phase model, and the crystallization process induced by thermal and/or deformation, as well as the microporosity of the PET structure that can be examined by the PALS method, are presented. After presenting the concept of a three-phase PET model, two more issues will be raised at the end of the literature review:

The microcavitation process (associated with free volume) of the PET microstructure as the main mechanism of lowering “the density of the non-oriented amorphous phase” during the deformation of PET samples [9].

The solid-state post-condensation (SSPC) as a chemical healing process [10] of PET microstructures (“chemical healing in semicrystalline linear polycondensate at a temperature close to melting includes the phenomena of transreaction, additional condensation, and diffusion” [11]) at temperatures slightly lower than the PET melting point and at a pressure close to vacuum (as a result of the reaction, a low-molecular by-product is formed, which is the molecule of ethylene glycol (EG), negatively affecting the further post-condensation process) [12]. The by-products of post-condensation processes as “small molecules diffusing through the polymer matrix are situated in the free volume, located in the amorphous zones of the material” [12].

The above two mechanisms are known in the literature but practically described only for a two-phase model in isothermal conditions. The research described in [7,8] shows that these two mechanisms occur simultaneously during the SBM process, overlapping, influencing each other, and limiting the negative impact of each of the two mechanisms on the physicochemical properties of the PET material, while the addition of rPET hinders the occurrence of this synergy. To the authors’ knowledge, taking into account the solid-state post-condensation phenomena during the microcavitation processes in the SBM process is a unique approach, and in the case of rPET, it is not yet described anywhere.

On the basis of the literature review, the conceptual model was developed, which combines solid-state post-condensation with microcavitation via the diffusion of the post-condensation product. At the same time, due to the lack of literature data on the mechanism of the post-condensation process occurring in the SBM process as well as on the mechanism linking the microcavitation process with the post-condensation process occurring in the SBM process, five additional hypotheses linking the microcavitation process with the post-condensation process was formulated, which will be the basis for further research.

It should be emphasized that the cavitation process is a broad and multi-disciplinary issue and is strongly dependent on the phase state of the material in which it occurs. In this review of the literature, the phenomenon of cavitation in a deformable semicrystalline polymer (being in a state significantly below the melting temperature) has been described, and other forms of cavitation were not analyzed (such as, for example, the phenomenon of hydrodynamic cavitation in a flowing fluid [13]).

## 2. Microstructure of PET Material

### 2.1. A Three-Phase PET Model

In contemporary research on PET material, the three-phase model of PET (mobile amorphous fraction (MAF), rigid amorphous fraction (RAF), and crystalline fraction) widely used [14,15,16], not the two-phase model of PET (crystalline regions embedded in an amorphous matrix). The three-phase model also takes into account the influence of the free volume on the macroscopic properties of the material, such as thermal or mechanical properties. Free volume parameters exert significant influence over various critical polymer properties, including gas permeability, selectivity, aging behavior, and mechanical strength [17]. Nonetheless, quantitatively estimating the size distributions of elementary free volume voids in polymers based solely on experimental data remains a complex challenge [17].

Studies by Lin et al. [18] and Michaels et al. [19] on oxygen permeability in semicrystalline PET at room temperature reveal that gas solubility decreases with the reduction in amorphous volume, but not in a straightforward manner. Lin et al. demonstrated that the observed decrease in oxygen solubility and diffusion coefficient in semicrystalline PET supports the three-phase model of these polymers. This model incorporates the rigid amorphous fraction (RAF) as a third phase, alongside the crystalline phase and the mobile amorphous fraction (MAF). RAF is characterized by thin amorphous layers located between crystal lamellae or between lamellar crystals and the mobile amorphous phase, with restricted molecular mobility compared to the normal amorphous phase. The research indicates that although the RAF volume fraction is smaller in melt-crystallized PET than in cold-crystallized PET, its specific volume and fractional free volume are substantially higher due to increased effective free volume. This explains the higher oxygen solubility found in melt-crystallized PET compared to cold-crystallized PET [18]. The RAF and MAF in PET can be quantitative analysis by PALS [20].

Olson et al. [20] wrote “The fractional free volume of RAF (…) has some memory of the equilibrium melt at the crystallization temperature, although the structure of RAF (…) varies with crystallization temperature”. These differences in RAF structure can be understood through PALS analysis and the three-phase model, which includes RAF with chain segments trapped between crystal lamellae during crystallization, along with the mobile amorphous fraction (MAF). Data on RAF and MAF volume fractions, derived from DSC and specific volume measurements, reveal the free volume characteristics of both RAF and MAF. Findings indicate that the fractional free volume of RAF, defined by hole number density and average hole volume, corresponds to the equilibrium melt state at the crystallization temperature [20]. However, the structure of RAF, as indicated by average hole volume, and hole number density shows significant differences between cold-crystallized and melt-crystallized samples [20].

In order to interpret the experimental data [7,8], it should be emphasized that, apart from nucleation caused by the presence of solid particles, there is also molecular nucleation, which is a particular case of primary crystallization concerning only a small part of the macromolecule after which further crystallization is preferred thermodynamically [21]. Each molecular order (crystalline phase) is associated with a similar shape (or conformation) of the polymer chain. A PET chain may be in a trans conformation or partially in trans and partially in gauche conformation. All conformations are related to the spatial structure of ethylene glycol segments [22,23]. The gauche conformations occur mostly in the amorphous phase of the material, while the crystalline phase consists only of the trans conformation [22]. The polymer model, in which the amorphous and crystalline regions (domains) are distinguished without distinguishing between different types of these structures or intermediate structures, is called a two-phase model. In the case of PET, the two-phase model, depending on the degree of crystallinity of the material, does not adequately describe changes in mechanical properties as a result of changes in temperature and/or deformation [22,24]. In the studies of amorphous and crystalline structures [22,24], it was found that there are two types of amorphous structures: mobile (more or less equal amount of gauche conformation and trans conformation) and rigid (significantly more trans conformation than gauche conformation), differing in the mobility of chain segments (chain stiffness resulting from a different amount of trans conformation, and the more trans conformation, the stiffer the amorphous structure). It was also found that there are various types of intermediate structures between the amorphous and crystalline structures, differing in the orientation of the chain in the non-crystalline phase (the so-called mesophases, resulting from different orientations of the amorphous phase, i.e., the nematic mesophase, smectic C mesophase, and smectic A mesophase [25]).

According to Boyd [26], the actual thermodynamic glass phase transition, in which the conformational entropy reaches zero (there are neither long-range movements nor short-range movements—the temperature of a discontinuity of the conformational entropy function of the material), is the transition temperature, the so-called T_2_. The T_2_ temperature, based on the Gibbs–Dimarzio theory, for PET is 52 °C lower than the glass transition temperature (T_g_), and for dry PET, T_2_ is 28 °C [26].

The crystalline, mobile amorphous, and rigid amorphous phases can be distinguished in the three-phase material model. An example of a three-phase model of PET structure (amorphous—rigidly amorphous—crystalline [27]) was proposed by Rastogi et al. [22], where the rigid amorphous structure arises as a result of the destruction (degradation) of the crystal structure (breaking long chains of similar conformation) caused by temperature (longtime heating) and/or deformation. The degradation of the crystalline material to the rigidly amorphous phase is unfavorable because the mechanical properties [22,27] and barrier properties [28] deteriorate (but only in the case of extensive deformation, in which large crystallites are destroyed and separated into smaller ones). Its potentially positive aspect may only be that the T_g_ increases for the material [24]. Detailed studies of the rigid amorphous phase have been described by Rastogi et al. [22]; however, the authors of the studies state that it is very difficult to isolate these structures and, therefore, very difficult to test their mechanical properties. They wrote that “rigid” amorphous structures have a lower density than “mobile” amorphous structures because, due to their stiffness, the chains of the “rigid amorphous” phase structure create a space with much larger voids than the mobile component. The analogous phenomenon of lowering the density of the oriented amorphous phase in relation to the density of the non-oriented amorphous phase was observed in the study of the SBM process for virgin PET (vPET) [6] and recycled PET (rPET) (Figure 4a in [7]).

**Figure 1:**
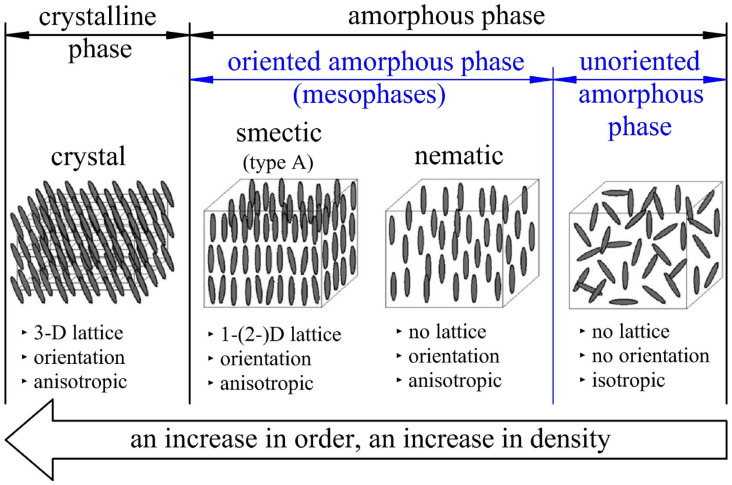
Schematic representation of the process of monomer orientation and formation of mesophases and then crystalline phase from the unoriented amorphous phase during temperature-induced crystallization and/or strain-induced crystallization with detailed features (crystal lattice, orientation, directional independence of physical properties (isotropy)) of individual phases and mesophases [29].

To put it in a few words, a three-phase model of PET microstructure represents the amorphous phase in two ways, depending on the ratio of the trans-to-gauche conformation of the PET macromolecule (MAF and RAF), the spatial arrangement of monomers relative to each other of PET macromolecules (unoriented and oriented mesophases), and the amount of free volume. The process of orientation of polymer monomers and formation of mesophases, and then of the crystalline phase, from the unoriented amorphous phase is schematically shown in Figure 1 [29].

### 2.2. The Crystallization Process Based on the Three-Phase PET Model

The material crystallized from the glass state (cold crystallization) is more likely to form rigid amorphous structures [22]. The analogous situations occur in the ISBM process, during reheating the preform in the heating furnace. An example of the influence of rigid amorphous structures on the reduction of the barrier of PET bottle walls to oxygen was described by Liu et al. [28]. Moreover, Rastogi et al. [22] prove that the three-phase model of PET, compared to the two-phase model, gives a lower degree of crystallinity (for the same measurements) and smaller crystal structures (in a material containing more “rigid” amorphous structures all dimensions of the crystalline structures are smaller). This is because compared to the three-phase model, the two-phase model captures the crystalline and “rigid” amorphous phases as one—crystalline.

Thermal (temperature-induced crystallization) and deformation (strain-induced crystallization) history, glycol content, the catalyst used, strain rate, and temperature are the factors that have the greatest impact on the crystallinity of virgin PET material [30]. It should be emphasized at the beginning that the thickness of the lamellae (crystalline structures) measured by SAXS strongly depends on the model used in the analysis. The calculated length of the isolated single lamella is approximately 80 nm, and its thickness is approximately 6 nm, while the lamella stack length was 5–20 nm [31]. It follows that single and isolated lamellae are much larger than stacked ones in strain-induced crystallization. This can be explained as follows. Upon formation, a newly isolated lamella can be relatively large due to its incomplete crystallization, resembling an intermediate structure akin to the smectic form. It undergoes fragmentation as it fully crystallizes during the drawing process [31] (it becomes “more perfect”—defects in crystallinity disappear). During stretching of the initially crystalline PET, the crystallinity of the material may decrease in certain areas as a result of a disturbed structure and increase in other places. It has been observed that the greater the initial degree of crystallinity of the material, the more the crystallinity decreases after deformation. An explanation of why this is so is provided by Nikolov et al. [32].

In simplified terms, it can be said that due to the morphology of the macromolecule, there are amorphous (non-oriented, mobile), crystalline, and non-crystalline phases. The amorphous phase comprises a high content of gauche conformation of the ethylene glycol segment, while the constrained non-crystalline phase is made up of chains rich in trans conformation of the ethylene glycol segment. The trans conformer shows different mobility in the amorphous and the ordered crystalline domains—conformational conversion to trans form—induce the subsequent crystallization (whereby the trans conformation may become a nucleus for crystallization [33]), which results in slower molecular motion [34]. Therefore, the more trans conformations in the non-crystalline phase, the less perfect the crystallites associated with diffusion problems, analogous to diffusion problems of long molecules [35]. This phenomenon has been previously described in terms of crystal growth regimes, where smooth-surface crystals are obtained at high crystallization temperatures. In contrast, highly imperfect crystals are obtained at low crystallization temperatures and intermediate ones where nucleation and growth or diffusion rates are comparable [35].

Furthermore, the transition to the more stable trans-conformation occurs before the formation of ordered crystallites. As crystallites develop, the fraction of the trans conformer is excluded from the PET amorphous phase, establishing the interface between crystalline and amorphous domains. The conversion to the trans conformer can be seen as the initial stage preceding crystallization [34]. For the material to crystallize, there must be crystallization seeds whose amount is determined by the thermal and mechanical history of the material.

It is reported that the thermal history of PET influences the morphological structure of rPET, even though the rPET granulate is fully melted [36] and that as a result of the melting of the structure, the crystalline structure of the granulate should completely disappear. However, it was found that the original degree of crystallinity, and even the fragmentation of the granules, has an impact on the quality of the bottle (along with the increase in granularity, most mechanical properties improve [37]). This is because, in order to crystallize, the material must include crystallization nuclei, the number of which is determined by the thermal and mechanical history of the material. Despite the disappearance of the crystalline structures during granulate melting, not all information about potential crystallization nuclei disappears (while the nucleation density and growth rate are higher in rPET than in PET [38]). For example, virgin PET (e.g., PET film obtained from virgin pellets) is very plastic (elongation at break > 200%), while polymer from post-consumer PET bottles (e.g., PET film obtained from regranulate) shows brittleness [39] (elongation at break > 10%). This is due to the differences in the crystallinity of materials, the presence of various inclusions in the recycled PET, and very often also significant differences in the viscosity and different mechanical and thermal history of virgin and recycled PET [40,41]. Changes in the trans conformation significantly impact the barrier properties of bottles. Studies indicate that permeability is higher in the gauche-amorphous phase compared to the trans-crystalline phase [23].

The ordered crystal structure is formed during annealing and deformation (above a certain degree of strain, referred to as the strain hardening parameter (SHP)—but only up to a certain strain rate depending on temperature) above the T_g_ and below the melting temperature. However, according to Rastogi et al. [22], too much deformation may cause degradation of the existing crystal structures to amorphous-like structures. The resulting structures show much greater chain stiffness (lower mobility of chain segments) than amorphous structures. The SHP value also depends on the method of deformation, and in the case of biaxial stretching, the SHP is lower than in the case of uniaxial stretching and is approximately 2.5:1 [42], while for uniaxial stretching, the SHP is 3.5:1 for the elongation ratio [42] (deformation approximately 250% of the original length). The SHP value is inversely correlated with the modulus of elasticity (Young’s modulus) and the tensile strength (the higher the stress at break, i.e., strength, the lower the SHP). It is directly related to the temperature and the stretching rate, and for lower temperatures or higher stretching rates, the SHP decreases [42]. It should be emphasized that the SHP is not directly related to the crystallization of PET but to the orientation of the amorphous phase.

According to Mahendrasingam et al. [43], three areas can be distinguished during isothermal uniaxial deformation at different rates and temperatures. Area 1 represents a high deformation rate at a relatively low temperature, where the start of crystallization occurs after the deformation is completed (but during the deformation, many smectic A structures are formed). The result is an extensive orientation of the strain-induced crystal structures (the crystal symmetry axis coincides with the deformation axis). Moreover, the crystallization rate depends on the deformation rate but is not dependent on the temperature—strain-induced crystallization, which can be approximated by the Avrami equation with the index n = 1 (primary crystal transformation). The orientation measurement obtained from FTIR is insensitive to changes in the deformation rate. Only in this area are the structures of the amorphous mesophase detectable. It is assumed that each nucleation process is preceded by the formation of an oriented amorphous mesophase (which is thermodynamically justified), which has been very well documented in the case of highly stretched PET fibers. It has been proven that the mesophase is formed during the stretching of PET film [44]. After the high-rate deformation is complete, the mesophase is transformed into a crystalline form. The mesophase structures were determined based on peak analysis of WAXS profiles for a wavelength of 10.2 Å. According to the information provided by Mahendrasingam et al. [44], mesophase structures are very difficult to observe and can be detected by WAXS under certain conditions (very high deformation rate at temperatures slightly higher than the T_g_ [44] or during deformation below the T_g_ from low rates [43]). At a deformation rate of 13/s and a temperature of 90 °C, they can only be observed for 0.2 s (these samples are very strongly oriented by the deformation) at the end of deformation with a deformation ratio of 3.7: 1 (for temperatures higher than 90 °C, this peak in the diffraction pattern of WAXS was not noted [44]). This first area corresponds to the deformation of PET occurring in the SBM process (with cold mold). Area 2 represents the average rate over the average temperature ranges, where the onset of crystallization occurs during the deformation, resulting in strain-induced oblique (inclined) crystal structures. Strain-induced crystallization can be approximated by the Avrami equation n = 1 (primary crystal conversion). However, the result is less than satisfactory, as there are some deviations from the primary conversion (this is due to the slower second crystallization process, which is most likely due to annealing during the deformation of the resulting crystals, which causes the reorganization of their morphological structure [43,45]). The relaxation of deformed chains in this area can be theoretically explained by Doi and Edwards’ (closing thin tube) and Gennes’ (creep concept) mechanisms, but it will not be discussed in more detail in this study. Area 3 represents a low deformation rate at a relatively high temperature where the strain-induced crystallization does not occur (no oriented crystal structures), and crystallization can be approximated by the Avrami equation with the exponent n = 3.

In summary, as the temperature rises, the strain-induced crystallization rate decreases due to the increased mobility of the polymer chain segments. Temperature influences the crystallization kinetics in normal temperature isotropic crystallization (from which isotropic spherulite structures are formed), but in the case of strain-induced crystallization, as the deformation rate increases, the significance of temperature decreases until its effect disappears completely.

### 2.3. The Crystal Nucleation Process of PET Material Based on the Three-Phase Model

**Figure 2:**
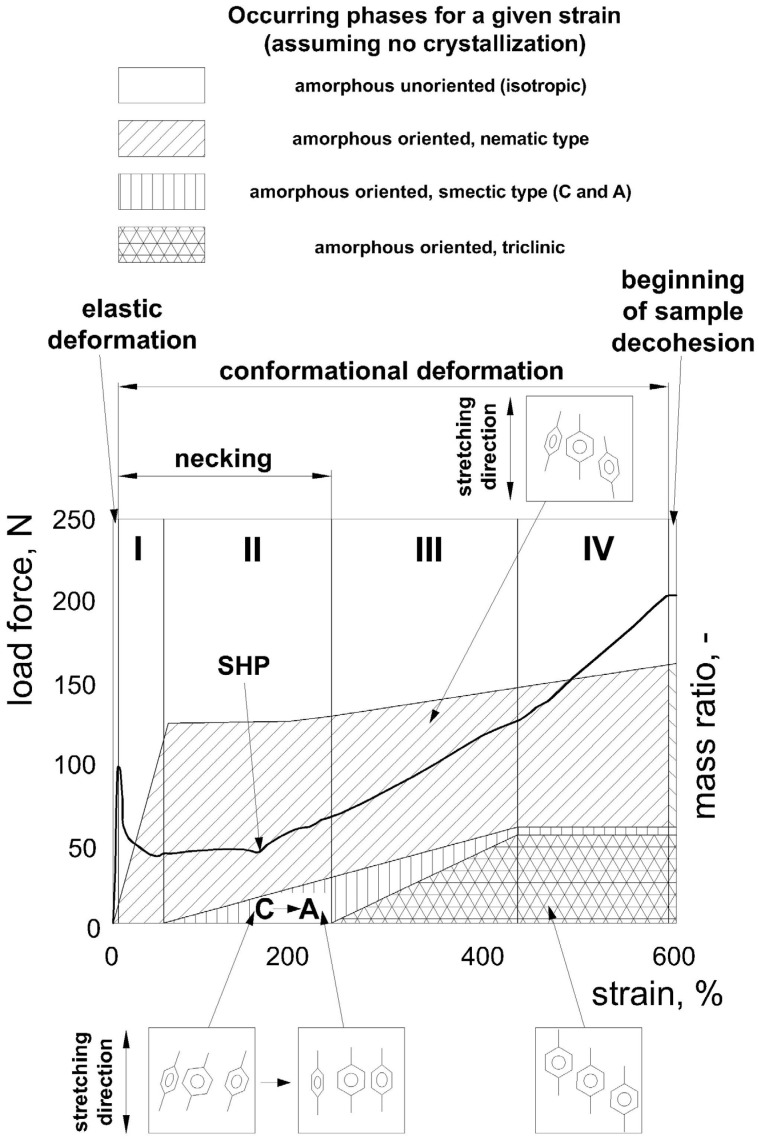
Schematic illustration of phase changes and division into four different stages of PET orientation during uniaxial stretching. The curve indicates the relationship between strain and load, while the differently hatched regions indicate the mass ratio of the occurrence of different phases: isotropic amorphous—non-oriented amorphous; oriented amorphous—nematic type; oriented amorphous—smectic type C and A; oriented triclinic amorphous (these are amorphous structures most similar to crystalline structures, and in the retraction process they change into a lamellar crystalline form) in a unit of sample mass and marking the area of neck formation [25]. The designation “C → A” denotes the transition of the smectic phase from type C to type A, and SHP means strain hardening parameter. The whole stretching range was divided into 4 ranges (I, II, III, IV), in which the changes of the oriented amorphous mesophase occur, i.e., I—sharp increase of the nematic, II—sharp increase of the smectic, III—slight increase of the nematic, and sharp increase of the smectic and triclinic, IV—slight increase of the nematic, and no change of the smectic and triclinic.

In summary, it follows from Kawakami et al. [25] that during uniaxial deformation, non-oriented amorphous structures (non-oriented amorphous phase) are oriented through the deformation force field to amorphous structures with significant order (intermediate, mesophase stages), and then to crystalline structures. The formation of mesophase phases (nematic or smectic) is observed on the stress–strain curve as the start of material strengthening (SHP). Figure 2 shows the influence of the mass content of mesophases (also strongly related to the orientation process) on the shape of the stress-strain curve during uniaxial stretching below the glass transition temperature (which allows the formation of a mesophase but prevents its transformation into a crystalline form).

In the case of phase transformations from amorphous non-oriented to crystalline, during the relative displacement of the chains with each other, the first intermediate stage is the nematic phase, i.e., the phase where segments of different entangled chains (or segments of one twisted chain) are arranged parallel to each other (longitudinal order), but they do not yet form a transverse order. In addition, they are oriented obliquely to the direction of deformation. Parallel segments are not yet on the same plane due to the relatively large distances between the chains.

With further elongation, the parallel chains of the nematic mesophase move relative to each other, which often leads to a change of conformation from gauche to trans (but unlikely vice versa) and increases the arrangement of the obliquely oriented chain segments in relation to the deformation direction (including polar groups and phenyl rings) to one plane, which leads to additional ordering, i.e., laterally skewed ordering (smectic C structures appear) [25].

Smectic structures are formed with two-dimensional (but not yet three-dimensional) order, i.e., longitudinal and transverse (forming non-crystalline bulk structures), but without conformational order (in these structures, the chain segments show both trans and gauche conformations). The formation of smectic C structures (with oblique order of phenyl rings) is the second intermediate stage of chain orientation during deformation. In the smectic C phase, there are more chains per unit of volume than in the unoriented or nematic amorphous phase, so the material density of the smectic C phase is higher. This ordering is stable (there are secondary interactions between the phenyl rings of different chains or segments of the same chain) during further deformation (up to a certain deformation above which they transform into smectic A structures) unless the strain rate or strain ratio is high enough to break the secondary bonds formed (if the strain rate and temperature are too high, plastic flow of amorphous entanglements is observed [46]).

Continuing to force the chains to move relative to each other causes the phenyl rings to move relative to each other, and from the oblique order (smectic C), the phenyl rings go to the perpendicular order (smectic A). Concurrently, the chain segments become oriented in the direction of deformation—as the deformation continues the smectic C phase changes into the smectic A phase [25]. This results in the fact that secondary interactions between the polar groups are added to the secondary interactions between the phenyl rings, but paradoxically, such a system is less stable because it introduces significant internal stresses between the chains. Due to this, the smectic A mesophase very quickly (even immediately) goes to the final structure of the oriented amorphous phase—the oriented triclinic structure [25] (dimensions of PET triclinic unit cell with a = 0.456 nm, b = 0.594 nm, c = 1.075 nm, α = 98.5°, β = 118°, γ = 112° [47], can be used for qualitative analysis in terms of perfection of crystalline phase and closeness to a fiber texture [40]).

It is worth mentioning that the density of the smectic A phase is comparable to that of the smectic C phase. The smectic A phase does not yet show conformational order (in the region of this phase, the chains have both trans and gauche conformations), the same as the smectic C phase [25].

The triclinic-oriented amorphous phase already has a three-dimensional order (longitudinal, transverse, and conformational) and contains only trans conformations [25]. Such a structure acts as a nucleus of crystallization, and if it reaches the required dimensions (determined thermodynamically [48]), it causes the surrounding chains to enlarge this structure—a lamellar crystal structure is formed. I.e., the resulting stable nucleus (with specific dimensions) starts the growth of the crystal structure, provided that sufficient energy is supplied (the T_g_ determines minimum energy) to the chain segments surrounding this nucleus. If the vibrations of the molecules caused by this energy become strong enough for the segments to slip out of the amorphous tangles of oriented chains or the non-oriented amorphous phase surrounding the nucleus and spontaneously change from the gauche conformation to the trans conformation, retraction processes may occur (chains moving closer to each other). Such extended chains with the trans conformation strongly interact with secondary bonds with the crystal nucleus and can connect with it through these secondary bonds—the crystal structure may grow due to retraction processes. The change of conformation from gauche to trans is induced thermodynamically because the free energy of the trans conformation is lower than that of the gauche conformation—the exception is the melting point at which the chains are supplied with such an amount of energy that the chains spontaneously move to a higher energy state, i.e., they move from trans to gauche conformation.

For temperatures close to the T_g_, the nucleation rate is maximum, which decreases with increasing temperature, while the growth rate of crystallites around the formed stable nuclei increases. At the crystallization temperature, the growth rate of crystallites is the highest, with a very low rate of formation of new nuclei. As the temperature increases, the growth rate of crystallites decreases, and the formation rate of new crystal nuclei also decreases until it drops to zero for both processes at the melting temperature [48].

After the recycling process of PET, the data from DMA and PALS suggest significant structural alterations in the amorphous region of PET, linked to a reduction in the size of free volume voids and the suppression of molecular mobility. Crystallization during degradation might be influenced differently by various factors. For instance, a decrease in molecular weight could enhance crystallinity, while reduced molecular mobility might suppress crystallization [49]. This transformation in the amorphous structure is likely a consequence of interactions between functional groups generated through degradation processes, such as hydrolysis or photo-oxidative chain scission during recycling and injection [50]. The weathering test conducted by Hagihara et al. [49] is anticipated to generate a substantial number of functional groups. For instance, hydrolysis could produce carboxylic acid and alcohol groups at cleaved chain ends. The interaction between these functional groups offers a plausible explanation for the observed reduction in free volume void size. Similarly, photo-induced cross-linking reactions may contribute to the reduction in free volume void size [49].

The decrease in molecular weight leads to greater molecular mobility, allowing for easier organization into crystalline structures. This behavior is also seen in PET, regardless of whether the reduction in molecular weight is due to photodegradation [51] or, hydrolysis [52] which involve different chain scission mechanisms.

### 2.4. PET Microstructure Analysis by the Use of Positron Annihilation Lifetime Spectroscopy (PALS)

PALS is an effective analytical method for PET [20,49,53,54,55], known for its capacity to assess the size of free volume voids in amorphous materials and imperfections within the crystalline domains of PET [56]. For over four decades, PALS has been employed as an experimental technique to measure local free volume [57,58,59,60]. PALS provides insights into cavity size within a material by correlating with the time between positron irradiation and positron annihilation. This technique also assesses the free space volume in polymers infused with nanofillers [61]. However, it should be remembered that crystallites in the polymer can be treated as nanofillers in the amorphous structure, in which nanofillers form covalent bonds with the amorphous matrix.

The concept of free volume, a fundamental element in polymer physics, originated during investigations into temperature-dependent viscosity and was initially applied by Doolittle to describe the viscosity of polymer melts [62]. The theoretical description of the PALS method has been discussed in detail in another article [7].

Extensive investigations have delved into the temperature-dependent behavior of free volume voids, emphasizing the pivotal role played by changes in “the hole expansion coefficient” during the glass transition, particularly as an experimental test of existing theories on free volume and free volume-regulated transport phenomena [54]. Thermal expansion is intrinsically linked to the magnitude of the distribution of free volume voids, encompassing size and concentration. Similarly, the physical aging process entails a time-dependent volume reduction, leading to corresponding alterations in the distribution of free volume voids. Consequently, changes in physical properties are intrinsically related to the free volume, molecular conformation, and entanglement within the polymer [54].

PALS is an effective tool for characterizing the “structural disorder in polymers” [58]. Notably, quantitative comparisons have been established [20] between characteristic parameters, such as intensity (I_3_) and lifetime (τ_3_) of the ortho-positronium (o-Ps) annihilation component of PALS, and the fractional free volume ($f_{v}$) of amorphous polymers, calculated using statistical mechanical theory. Specifically, I_3_, reflecting the probability of o-Ps formation, serves as an indicator of the density of free volume voids, while τ_3_ can be linked to the void radius (R) and, consequently, the void volume $v_{f}=\left(4\cdot \pi /3\right)\cdot R^{3}$. Therefore, “fractional free volume” $f_{v}$ of amorphous polymers $f_{v}=C\cdot I_{3}\cdot v_{f}$, where C is a constant specific to each polymer [20]. However, constant C is estimated through studies on a large number of polymers, and this “equation for the determination of nanohole density or fractional free volume is extensively discussed in the literature” [61].

In the realm of porous and amorphous systems, PALS emerges as a powerful tool capable of investigating cavities spanning from fractions of a nanometer to several tens of nanometers [63]. However, some authors suggest that positron annihilation exhibits sensitivity to micropores (<2 nm) but lacks the capability to detect mesopores (2–50 nm) [64]. This specificity arises from the limited diffusion length of positronium within a polymer and the non-uniform distribution of mesopores within heterogeneous systems [64]. Consolati et al. [63] provide a comprehensive introduction to PALS, with a particular focus on readers unacquainted with this methodology, emphasizing its experimental aspects.

## 3. Cavitation and Solid-State Post-Condensation of PET Material

### 3.1. Cavitation and Microcavitation Effect in PET Material

Generally, a distinction should be made between the cavitation process—referring to the formation of cavitation pores that accompany the uniaxial stretching of most semicrystalline polymers—and the microcavitation effect, as defined by Equation (1) in [7], which involves the presence of free volume pores within the amorphous phase. However, research on cavitation process during deformation has much richer literature than microcavitation, which is understood as an increase in the porosity of the amorphous phase. We must strongly emphasize that no literature has been found proving that the direct application of the analogy between cavitation and microcavitation is correct for the SBM process. However, some assumptions for the proposed mechanism of post-condensation occurring in microcavitation areas in the SBM process are supported by the literature examining cavitation issues (further development and research will be carried out to check the adopted assumption in [7]). The problem with the analogy between cavitation and microcavitation for the SBM process results from the complexity of the microstructure transformation phenomena occurring in the SBM process (as well as the thermodynamic complexity of the SBM process itself). I.e., in the SBM process, initially amorphous material is deformed at elevated temperatures at high deformation rates, resulting in the crystallization of the polymer (and there would be no problem with the analogy of microcavitation to cavitation). However, the analyses carried out on the SBM process (the research results are shown in Tables S4, S5, and S6 in [7]), described and interpreted in detail in [8], show that the entire process is much, much more complex. For example, in the SBM process, in addition to crystallization (understood as all processes related to the crystallization process, such as the orientation of the amorphous phase or the formation of crystallization nuclei in the self-nucleation process), there is also a change in the shape of the free volume pores in the amorphous phase. The parameter defined as microcavitation effect (Equation (1) in [7]) is interpreted as a change in the porosity of the material at the level of free volume and not the formation of cavitation pores observed, for example, when stretching polypropylene (PP) [65] or high-density polyethylene (HDPE) [66,67]. The measure of this parameter is the change in the density of the amorphous phase. It should be emphasized that the τ_3_ value from PALS analysis (Table S6 in [7]) for bottles is shorter than in preforms (this means that the average lifetime of the ortho-positronium and, therefore, the average pore size of the free volume is also smaller). In contrast, fitting uncertainty for τ_3_ time (Table S6 in [7]) and dispersion of ortho-positronium lifetime (σ_3_, Table S5 in [7]) is greater for the bottle material than the preform. Such an effect can be interpreted as a change in the shape of the free volume pores (from statistically spherical) in the case of materials with an oriented amorphous phase. Then, the annihilation of ortho-positronium along the shorter axis of the ellipsoidal pores of the free volume is more likely than along the longer one (so the technique of measuring the dimensions of the free volumes using the PALS method is sensitive to the shape of the free volumes themselves), and concurrently, the density of the amorphous phase (as a measure to calculate by Equation (1) microcavitation effect in Table S4 presented in [7]) decreases. However, the microcavitation testing technique based on the density of the amorphous phase (defined by Equation (1) in [7]) is not sensitive to the shape of the pores [9].

**Figure 3:**
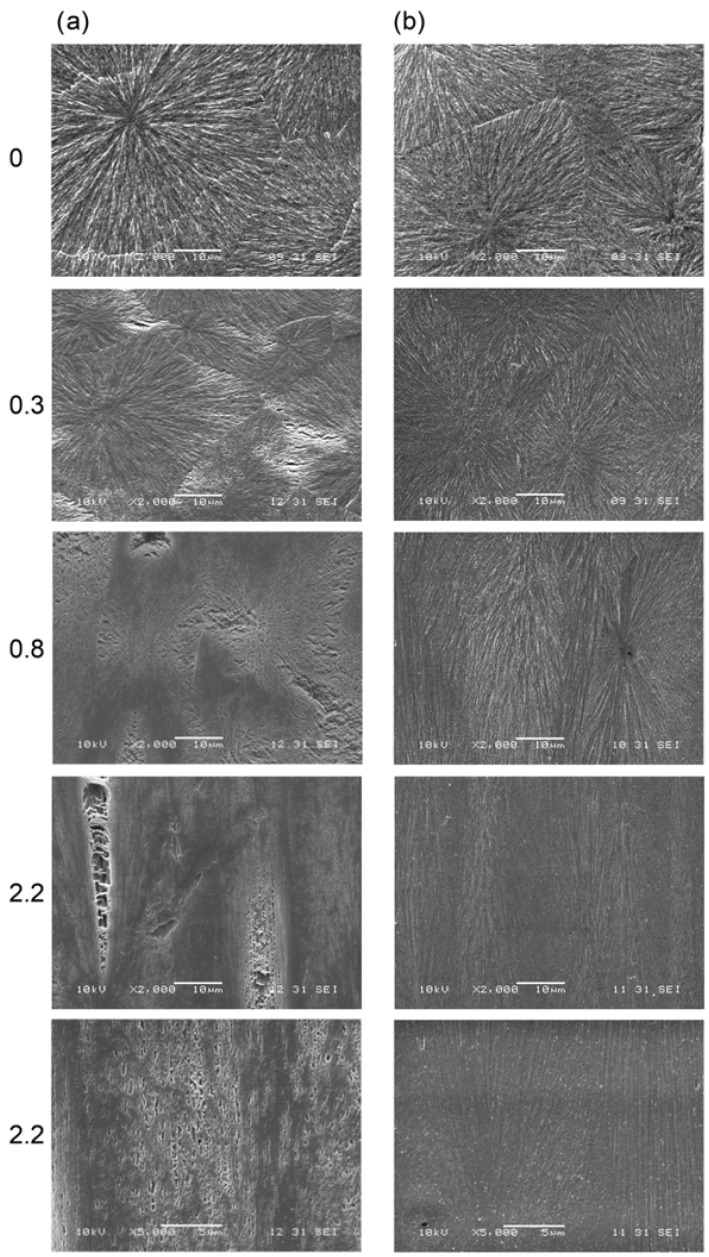
SEM microphotographs of cavitating (**a**) and saturated with chloroform non-cavitating polypropylene samples (**b**). Microphotographs show the etched longitudinal section of the sample. Morphological details unraveled by cryoultramicrotomy followed by etching. The numbers on the left of microphotographs correspond to the local strain of samples. The direction of deformation: vertical (relative to the microphotographs shown) [86].

The literature on the cavitation process during stretching is quite extensive and covers polymers such as polypropylene [65,66,67,68,69,70,71,72,73,74,75,76,77], polyethylene [78,79], or polybutene [80,81]. It is well established that cavitation behavior is impacted by a range of factors, which can be categorized into two main groups. The first group comprises experimental factors, such as temperature and deformation rate, with lower temperatures or higher rates generally promoting void formation. The second group involves the polymer’s microstructure, such as lamellae thickness, crystal form, and the state of the amorphous phase. Depending on specific processing conditions, polymer processing can produce various microstructures and morphologies in the final product [82,83,84]. Thus, comprehending how microstructure affects void formation is crucial for establishing a structure-properties relationship. Based on the above-mentioned literature, it can be summarized that:

1. During film-sample deformation, mesophase transformations to higher-density structures lead to the sample’s volume contraction, possibly forming voids in the sample.
2. The porosity of stretched films, driven by the presence of voids, increased as the drawing ratio rose.
3. Cavitation occurs sooner with a higher number of tangential lamellae within a spherulite. Conversely, reducing the number of tangential daughter lamellae accelerates void formation.
4. Cavitation emerged at the spherulite boundaries or in their equatorial regions, where the lamellar crystals are oriented perpendicularly to the tensile direction.
5. Annealing can accelerate the onset of cavitation [75]. In annealed samples, cavitation behavior was greatly intensified due to an increase in crystal thickness and a rise in stress concentration sites. This behavior may explain the reduction in pressure resistance observed in PET bottles produced in a hot mold during the SBM process, as noted in another work by the authors [6]).
6. The presence of thinner lamellae generally inhibit void formation due to their higher density of tie-molecules. This denser network facilitates more effective load transfer to the lamellae, promoting their plastic deformation over cavitation within the amorphous phase, as observed in research on polybutene [81].
7. Samples with lower molecular weight exhibited stronger cavitation due to fewer entanglements in the amorphous phase.
8. When crystallite movement in the amorphous phase is restricted at relatively low temperatures, cavitation increases as the sample is stretched parallel to the lamella orientation (based on research of PP) and void forms before fragmentation and reorientation of microstructure—with rising temperature during stretching, void size diminishes.
9. Introducing a low molecular weight modifier into the free volume pores of the amorphous phase results in reduced intensity (“the type of liquid is not relevant, except that it should not dissolve polymer crystals” [85]), or complete elimination, of the cavitation phenomenon. SEM microphotographs of cavitating (a) and saturated with chloroform non-cavitating polypropylene samples (b) are shown in Figure 3 [86].
10. Initially, during stretching, voids elongate perpendicular to the stretching direction and subsequently reorient along the stretch axis. In the early stages, incorporating a nucleating agent (NJS) has minimal impact on void size. However, at later stages, voids along the stretching direction in iPP/NJS composites grow rapidly.
11. Cavitation occurs in various semicrystalline polymers when they are stretched uniaxially above their glass transition temperatures. Void formation is typically affected by the polymer’s morphology, including lamellae thickness, orientation, and the microstructure of the amorphous phase. During stretching, void sizes vary according to local strain levels [68].

Cavitation occurrence in semicrystalline polymers (SCPs) varies due to the interplay between amorphous phase breakage and lamellar crystal shearing. If the stress required to initiate cavitation under tensile loading is lower than that needed for plastic deformation of lamellae, substantial void formation occurs between the lamellae. SCPs with thick, well-developed crystals form cavities or voids, requiring higher critical stress for crystal shearing than for cavitation activation [87]. Conversely, SCPs with thinner or more defective crystals, or those with lower molecular weight, tend to prevent cavitation. Studies have shown that a lower number of entanglements and tie molecules in the amorphous phase encourages cavitation occurrence [87]. In contrast to low molecular weight materials, the amorphous phase of crystalline polymers exhibits remarkable cavitation resistance, typically 10–20 MPa, attributed to amorphous layer confinement between crystalline lamellae and macromolecular chain entanglements, absent in low molecular weight liquids [88]. Importantly, processes like lamellar fragmentation and crazing remain poorly quantified to date [87].

As a result of the cavitation process in the amorphous phase, the temperature of the PET material increases. Ronkay described this mechanism [89] as follows: “When the stress increases, the polymer cavitates with acoustic emission. The material between cavities exhibits the fibrillar structure, and heat is generated. However, due to cavitation, the heat conductivity of PET decreases considerably, so the local temperature increases abruptly” (the temperature on the sample surface increased from 22 °C to approximately 74 °C, i.e., by approximately 50 °C, wherein the temperature values inside the specimen could be considerable higher).

When SCPs undergo tensile deformation, rupture often proceeds through “cavitation-induced damage processes” involving “void nucleation, growth, and coalescence activated by chain scission or disentanglement” [87]. Cavitation is thus recognized as a primary damage mechanism in SCPs. Quantitative descriptions of cavitation in SCPs employ damage parameters like volume strain or porosity, based on theories such as mechanics of porous media (MPM) or continuum damage mechanics (CDM). However, understanding damage mechanisms in SCPs under different loading conditions, particularly compression, creep, and fatigue, remains incomplete [87]. In general, semi-crystalline PET material can be simplified into four geometric shapes: lamellar, acicular, discoid, and spheroidal, although their low sphericity poses a challenge to treating them as spheres [90].

Damage in the crystalline phase involves “lamellar shear, fragmentation, and chain unfolding”. Cavitation damage in the amorphous phase influences the deformation and damage behavior of crystalline components, and vice versa. There is a hypothesis that cavitation-induced lamellar fragmentation is triggered by the release of 3D stress, promoting cavity formation [87]. Fragmentation leads to the formation of a fibrillar structure observed using SAXS (small-angle X-ray scattering), WAXS (wide-angle X-ray scattering), and TEM (transmission electron microscopy). The microstructure of SCPs’ amorphous and crystalline components critically determines their deformation and damage mechanisms, influencing the likelihood of cavitation. SCPs with higher crystallinity and thicker crystals tend to suppress dislocations, favoring cavitation occurrence [87].

### 3.2. Solid-State Post-Condensation of PET

In the case of PET, molecular weight and dispersion changes during mechanical processing can be largely remedied [91]; the presence of esters in the PET macromolecule makes it possible to moderate changes in these properties through post-condensation in the solid state or the addition of chain extenders. An increase in the molecular weight of PET as a result of post-condensation processes was observed even during PET combustion [92]—these processes improve its flame retardancy.

The solid-state post-condensation process, also known as chemical healing, for PET has been thoroughly understood qualitatively [10,93]. However, quantitative understanding remains a challenge, with the literature often presenting conflicting data and models, especially for semi-crystalline polymers and under non-isothermal conditions [12]. The complexity of solid-state post-condensation (SSPC) reactions largely hinges on the interaction between diffusion processes: the removal of by-products from particles and the reaction of reactive end groups with each other. Factors such as temperature, duration of SSPC, particle size, and environment significantly influence the molecular weight increase in polyesters [10]. Polyester exchange reactions typically occur in the molten state (approximately 5–10 °C above melting temperature) [10]. However, ester interchange reactions of PET can also occur 20–30 °C below its melting point, specifically in the solid state, catalyzed by free carboxylic end groups [10]. Diffusion, particularly chain interpenetration, is primarily considered in amorphous regions, although chemical interactions can occur even within crystalline regions [10].

The importance of solid-state condensation is expected to grow steadily due to increasing demands for packaging materials. In developing countries, inadequate packaging leads to spoilage of 50% of food, contrasting with 2–3% in the UK [10].

Based on the literature data, Gantillon et al. [12] discussed the cause-and-effect sequence that conceptually explains the phenomenon of post-condensation in the solid state in semicrystalline polymers at temperatures lower than the melting point. The idea proposed in this work of the post-condensation phenomenon occurring in the SBM process (i.e., at temperatures higher than the T_g_ but significantly lower than the melting temperature) is mainly based on the idea described by Gantillon et al. The following is the most important information from the literature review conducted by Gantillon et al. [12], necessary to conceptually develop the phenomenon of post-condensation in the solid state with simultaneous occurrence of microcavitation phenomenon in PET material in the SBM process.

In the two-phase model, reactions progress incrementally within the amorphous regions of semicrystalline polymers. Even the melting process of the crystalline regions in these polymers is influenced not only by the thickness of the crystals but also by structural constraints within the non-crystalline regions [94]. By-products, end groups, and low molecular weight oligomers are selectively excluded from the crystalline regions. According to this model, the diffusion of end groups occurs predominantly within the amorphous phase through the movement of oligomers, terminal segments, or through exchange reactions. Functional groups remain at the ends of formed macromolecules, capable of further reactions under appropriate conditions [10]. This facilitates the proximity of reactive end groups, thereby enhancing their reactivity [95]. The number of end groups of macromolecules varies based on the polymer mass and its distribution, making it a distinctive characteristic of each material, whether it is pure or recycled.

In solid-state reactions, polycondensation or transesterification occurs when chain ends diffuse toward each other, and ethylene glycol (EG) diffuses out of the solid matrix into the continuous medium [12]. Crystalline regions impede EG diffusion, while free areas aid diffusion from the polymer matrix. Small molecules diffuse in the free volume, mostly in amorphous zones—EG diffusion coefficient through the polymer matrix and any present pores influence polycondensation rate, alongside temperature, PET matrix crystallinity (amount of crystalline material, size, and shape of crystalline occlusions, etc.), solid-state pore structure (size and tortuosity of the pores), and inert gas concentration [12].

In ‘chemical’ diffusion involving chain end mobility, reactions become complex. In the melt phase, chain end groups distribute homogeneously, leading to rapid polycondensation, and the observed rate constants of polycondensation are independent of the concentrations of hydroxyl and carboxyl groups—in this scenario, the polycondensation rate will be governed by the diffusion rate of the chain ends towards one another rather than the diffusion rate of EG. However, in the solid state, the distance between chain end groups increases, potentially slowing the reaction. Despite this, ester interchange reactions enable polycondensation to proceed, driven by the migration of active species along the chain backbone. Ester interchange reactions drive polycondensation by occurring randomly among various reactive end groups (acids, esters, or alcohols) and ester groups within a macromolecule’s crystalline occlusion. Additionally, active species likely migrate along the chain backbone to sustain a suitable reaction rate in the solid state [12].

Increasing temperature enhances the mobility of reactive chain ends, accelerating the polycondensation rate. Moreover, higher catalyst concentration and accessibility within the polymer’s amorphous regions facilitate transesterification, even at lower temperatures. Crystallization excludes defects like catalyst-bound chain ends, elevating catalyst concentration in the amorphous phase, notably at crystal surfaces [12] (i.e., in places where microcavitation phenomena occur to a large extent [6,9]). “Impurities”, including “chain ends, low molecular weight oligomers, branches, additives, catalyst residue, and any other molecules that do not possess the symmetry needed to form crystals”, concentrate outside polymeric crystal lamellae in the solid state. This concentration in amorphous regions with high molecular mobility sustains reaction rates. Continuous chain growth and interchange reactions promote ongoing reorganization and crystallization during the reaction process [12].

## 4. General Conclusions from Literature Review

A review of the literature shows that in the SBM process, post-condensation of the PET can occur in the solid state, within the amorphous regions of material, despite the preforms being heated well below the melting point. This is evidenced by the intrinsic viscosity and PALS tests of the preform and bottle material at their respective points, which are presented in Tables S4, S5, and S6 in [7]. Moreover, the research presented in article [8] shows that the addition of rPET hinders the occurrence of post-condensation processes.

It is a commonplace misconception in the industry that in the SBM process, the length of the PET chain can only be shortened as a result of mechanical rupture. However, this is unlikely because the breaking stress of the PET chain is gigantic (theoretically the breaking stress of covalent carbon-carbon bond of a two atom molecule is approx. 360 GPa, but the strength of –C–C– bonds in a polymer chain is practically 60–100 GPa [96]). Moreover, it was shown that during the processing of PET in the SBM process, post-condensation processes in the solid state may occur, resulting in an increase in the molecular weight of the PET chain, which is measured by the increased value of the intrinsic viscosity of the bottle material relative to the preform material [7,8].

The literature describing the mechanism of post-condensation process in PET material in the SBM process has not been found. Nonetheless, the above literature review reveals that in the SBM process, both microcavitation processes and the solid-state post-condensation (SSPC) processes can occur in the PET material, which was confirmed in the studies described in another works [6,7]. Concurrently, due to the lack of data in the literature on the mechanism of the post-condensation process occurring in the SBM process, as well as on the mechanism linking the microcavitation process with the post-condensation process occurring in the SBM process, five additional hypotheses linking the microcavitation process with the post-condensation process can be formulated, with the first two being contradictory due to the influence of rPET content on microcavitation process. These first two were verified in the study described in other articles [7,8]. The remaining three hypotheses will be tested in the future.

**Hypothesis** **1.** *The addition of rPET reduces the occurrence of microcavitation and, consequently, post-condensation because the crystallites with the addition of rPET are less perfect, and due to this, microcavitation decreases as well as the efficiency of the post-condensation process in the vicinity of the volume of voids (“cavitation is prevented in SCPs possessing thinner or more defective crystals or lower molecular weight” [87]).*

**Hypothesis** **2.** *Or, the addition of rPET increases the trans conformation content in the amorphous phase (which increases the stiffness of this phase), which reduces the mobility of the free ends of PET macromolecules, reducing the efficiency of the PET solid-state post-condensation process inside, but without reducing the microcavitation effects. For increasing amount of the rigid amorphous structures the T_g_ increases for the material [24] and the shift of T_g_ towards higher values hinders the migration of macromolecule fragments/chain ends (this should further hinder/inhibit the post-condensation process). This hypothesis was formulated based on the shift in T_g_ values in the preforms described in related research articles (see Table S4 in [7]). However, the observed changes are relatively small*.

**Hypothesis** **3.** *The release of heat as a result of intermolecular friction and changes in confor-mation from gauche to trans of the PET chain in the vicinity of the microcavitation area (which significantly reduces the thermal conductivity coefficient) can significantly increase the temperature of the PET material in the vicinity of the micro-cavitation area even close to the melting point, which may result in post-condensation of PET (especially since the intrinsic viscosity tests clearly indicate this, what was described in related research articles (see Table S4 in [7])). At the same time, the deformation process in the SBM process leads to a significant reorganization of the material structure at the molecular level (bringing the free ends of the PET chains closer to each other), which also positively affects the efficiency of post-condensation. Both factors (i.e., a significant increase in the local temperature and the kinematically forced deformation field) are important as elements promoting the movement/migration of macromolecules and, therefore, chain ends, which in turn leads to post-condensation observed in the intrinsic viscosity*.

**Hypothesis** **4.** *The released by-products from the post-condensation process of the PET material diffuse into the free spaces created as a result of the microcavitation of the amorphous phase, inhibiting the increase in the microcavitation volume (microcavitation volumes do not propagate into cavitation volumes) and thus hindering the disruption of crystallites during their rotation in the amorphous phase*.

**Hypothesis** **5.** *The lower the temperature of heating the preforms in the SBM process, the initially stronger the microcavitation processes, which induce post-condensation processes, as a result of which small-particle products are released, filling the free microcavitation volumes, thus limiting further microcavitation processes, and favoring the orientation of the microstructure of the material. This explains why, despite low preform heating temperatures, higher pressure strength of the bottles is achieved, and that the addition of rPET reduces the pressure strength of the bottle because it hinders the occurrence of post-condensation phenomena—as a result of which, during the microcavitation processes, the post-condensation process occurs with lower efficiency in microcavitation areas. As a consequence, much higher-pressure strengths of bottles are observed for low-heated preforms than for bottles made of more highly heated preforms*.

**Figure 4:**
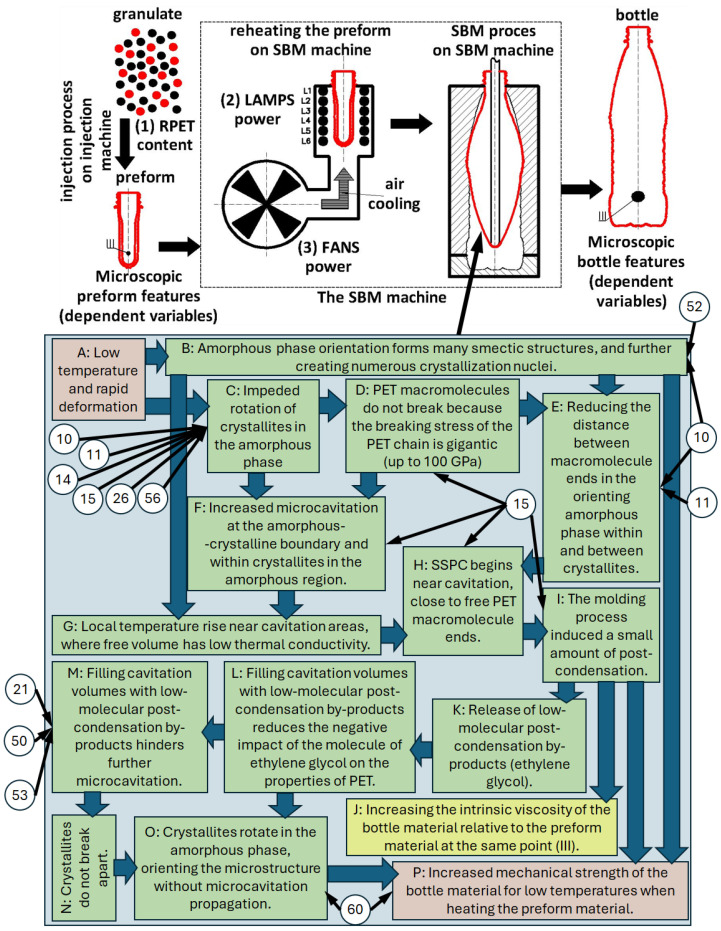
Schematic representation of the interaction potentially occurring in the low-temperature SBM process between the phenomenon of microcavitation and the phenomenon of post-condensation in the solid state and its impact on the microscopic and macroscopic properties of containers produced in the SBM process. The letter designations (A–P) are supported by the literature (description in the text), while the numerical designations (10, 11, 14, 15, 21, 26, 50, 52, 53, 60) are described in the related research work [8] and correspond to the notes in figures of the test results [8].

**Figure 5:**
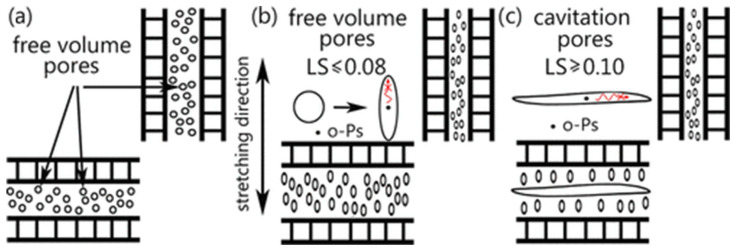
Nanostructure of the amorphous layers (**a**) and its deformation-induced changes before (**b**) and after (**c**) the initiation of the cavitation phenomenon (LS—local strain). Influence of pore anisotropy (free volume and cavities) on pick-off annihilation of o-Ps in PALS (red arrow) [9].

All the above five hypotheses are interconnected by microcavitation and post-condensation phenomena in the solid state, as schematically illustrated in Figure 4. A schematic explanation of the SBM process with the selection of independent variables accepted in related research articles [7,8] (rPET content, heating lamp power, cooling fan power, and SBM process treated as a whole) and dependent variables as microscopic features of the preform, and the bottle is also shown in Figure 4 (the numerical designations (10, 11, 14, 15, 21, 26, 50, 52, 53, 60) are described in the related research work [8]). The diagram shown in Figure 4 uses the designations A–P, where:

A: Relatively low temperature and very quick deformation of the PET material during the SBM process [97] (stiffer amorphous phase)—the temperature of the preform, depending on the shape and thickness of the side wall of the preform and the shape of the container intended for cold fill, ranges from 105 °C to 128 °C [1].
B: Occurrence of the orientation of the amorphous phase with the formation of a large number of smectic structures and thus a very large number of crystallization nuclei [25] (no crystallization of the PET material during rapid deformation at relatively low temperatures), especially in biaxial stretching [42]—immediate crystallization induced by the deformation of the material occurs after the fast deformation process is completed [43,44].
C: Impeded rotation of crystallites in the amorphous phase [68,98].
D: PET macromolecules do not break because the breaking stress of the covalent carbon-carbon bond in PET chain is gigantic (the strength of a polymer chain depends on the strength of –C–C– bonds, practically 60–100 GPa [96]).
E: Shortening the distance between the free ends of PET macromolecules in the orienting amorphous phase inside the crystallites and at the boundary of the crystalline and amorphous phases [25]. Free ends of PET macromolecules emerging from the crystallites are not entangled with each other [12], which occurs only in the amorphous phase, which may favor post-condensation of PET macromolecules in free volumes (vacuum) created as a result of microcavitation between the lamellas in the crystallite, as well as at the boundary of the crystalline and amorphous phases.
F: Increased microcavitation at the boundary of the amorphous and crystalline phases and inside the amorphous regions [98,99].
G: Local increase in temperature (e.g., as a result of intermolecular friction [46,89] or as a result of a change in the conformation of the macromolecule from gauche to trans [100]), whereas for very rapid deformation the heat transfer conditions are almost adiabatic, so all the released heat of crystallization (which occurs after completion of deformation at high deformation rates) will increase the sample temperature [101]) in the vicinity of microcavitation areas (the free volume is characterized by a very low thermal conductivity coefficient) [89]
H: Initiation of the post-condensation process in the solid state near the microcavitation area in the vicinity of the free ends of PET macromolecules near crystallite boundaries [12].
I: The molding process induced a small amount of post-condensation, which can be determined by (J).
J: Increasing the intrinsic viscosity of the bottle material relative to the preform material at the same point (Figure 4b in [7]).
K: The release of low-molecular by-products of the post-condensation process (molecule of ethylene glycol) [12].
L: Filling free microcavitation volumes with low-molecular products of the post-condensation process, reducing the negative impact of the molecule of ethylene glycol on the properties of PET [12].
M: The process of filling empty microcavitation volumes with low-molecular products of the post-condensation process hinders further microcavitation processes [88], as a result of which the microcavitation area does not propagate in the amorphous phase inside the crystallite, and at the border of the crystalline and amorphous phases [77] (the reduction of the dimensions of the free volumes results in a decrease in their susceptibility to ellipsoidization during polymer deformation as a consequence of what microcavitation volumes do not propagate into cavitation volumes [9]—the mechanism of the influence of deformation on the ellipsoidization of the free volumes, their approaching each other, and consequently their propagation into the cavitation volumes is schematically shown in Figure 5 [9]), increasing the strength of the amorphous phase inside the crystalline phase (by filling free volume pores, their average size decreases, increasing the strength of the amorphous phase, which in turn hinders the process of creating cavitation pores—higher stresses are required to create them and stabilize such pores) [77].
N: Crystallites do not break apart (filling the free volume pores and reducing their average size increases the strength of the amorphous phase [77]).
O: The crystallites rotate in the amorphous phase, orienting the microstructure without the propagation of microcavitation due to the free volumes being filled with low-molecular products of the post-condensation process.
P: Increased mechanical strength of the bottle material for low temperatures when heating the preform material [6,30].

The technology described in the article concerns SMB (stretch blow moulding) process from preforms. This technology is mainly used to produce packaging from PET and PP (polyethylene) materials. In the beverage industry, the dominant material is PET. This is due to the much better mechanical properties of PET packaging, significantly better barrier properties (CO_2_ and oxygen permeability) and much better visual quality of packaging. PP bottles are practically not used to store carbonated water (beverages) [102,103,104].

In addition to the preform blowing technology, single-stage blowing technology is widely used, i.e., a “sleeve” is formed directly from the plasticized granulate, which is formed into the shape of the bottle in the blowing process. This technology is used especially in the cosmetics and the pharmaceutical and chemical industries and mainly concerns PP. Additionally, something that is especially important today from the point of view of the closed-loop economy, is minimizing the amount of material introduced to the market. In the case of still water, we can find 500 mL PET bottles weighing up to 9.5 g on the market. In the case of PP, 500 mL bottles weighing less than 20–22 g are not currently found.

The development direction of blow molding technology, especially in the beverage industry, is constantly moving towards reducing the weight of the packaging used, increasing its mechanical strength and full control of the technological process (blow molding process) [105,106,107,108]. These activities are related not only to reducing the costs of manufacturing this type of packaging, but also to environmental protection (smaller amount of material introduced to the market). An interesting direction of further research related to the topic of the article is the possibility of answering the following three questions:

1. Is it possible to control the post-condensation process and the microcavitation phe-nomenon during the blow molding process?
2. What effect do post-condensation and microcavitation have on the mechanical strength of blow molding packages?
3. Do the described phenomena also occur in other blow molding plastics, with particular emphasis on PP (Polypropylene)?

Knowing the answers to the above three questions could provide an impulse for further work on the cost optimization of blow molding packages.
